# Federated learning-based multimodal approach for early detection and personalized care in cardiac disease

**DOI:** 10.3389/fphys.2025.1563185

**Published:** 2025-04-23

**Authors:** Sultan Alasmari, Rayed AlGhamdi, Ghanshyam G. Tejani, Sunil Kumar Sharma, Seyed Jalaleddin Mousavirad

**Affiliations:** ^1^ Department of Information Systems, College of Computer and Information Sciences, Majmaah University, Majmaah, Saudi Arabia; ^2^ College of Technology and Business, Riyadh Elm University, Riyadh, Saudi Arabia; ^3^ Department of Information Technology, Faculty of Computing and Information Technology, King Abdulaziz University, Jeddah, Saudi Arabia; ^4^ Department of Research Analytics, Saveetha Dental College and Hospitals, Saveetha Institute of Medical and Technical Sciences, Saveetha University, Chennai, India; ^5^ Department of Industrial Engineering and Management, Yuan Ze University, Taoyuan, Taiwan; ^6^ Department of Computer and Electrical Engineering, Mid Sweden University, Sundsvall, Sweden

**Keywords:** cardiac diseases detection, federated learning, attention-based feature fusion, SGD-DNN, deep neural network

## Abstract

**Introduction:**

Heart disease remains a leading cause of mortality globally, and early detection is critical for effective treatment and management. However, current diagnostic techniques often suffer from poor accuracy due to misintegration of heterogeneous health data, limiting their clinical usefulness.

**Methods:**

To address this limitation, we propose a privacy-preserving framework based on multimodal data analysis and federated learning. Our approach integrates cardiac images, ECG signals, patient records, and nutrition data using an attention-based feature fusion model. To preserve patient data privacy and ensure scalability, we employ federated learning with locally trained Deep Neural Networks optimized using Stochastic Gradient Descent (SGD-DNN). The fused feature vectors are input into the SGD-DNN for cardiac disease classification.

**Results:**

The proposed framework demonstrates high accuracy in cardiac disease detection across multiple datasets: 97.76% on Database 1, 98.43% on Database 2, and 99.12% on Database 3. These results indicate the robustness and generalizability of the model.

**Discussion:**

Our framework enables early diagnosis and personalized lifestyle recommendations while maintaining strict data confidentiality. The combination of federated learning and multimodal feature fusion offers a scalable, privacy-centric solution for heart disease management, with strong potential for real-world clinical implementation.

## 1 Introduction

Heart disease or cardiovascular disease (CVD) refers to conditions, which involve heart disease (HD), coronary artery disease (CAD), heart attack, and heart failure. Timely intervention and an accurate diagnosis of heart disease are a must as identification at early stages can avoid such severe complications and bring the patients back to a good life (Alqahtani et al., 2022). It is very challenging to diagnose heart disease because the disease is complicated and manifests itself in different ways in different patients (Kavitha et al., 2021; Abubaker and Babayiğit, 2022). Further, conventional diagnostic techniques can be laden with human error, which may lead to either false positive or false negative results. False early detection results in unnecessary treatments or missed opportunities for vital care (Saboor et al., 2022).

The prevalence of heart disease is still alarmingly high and affects millions of people worldwide. The World Health Organization claims that CVD are the leading cause of death in the world, with almost 18 million people dying from them every year (Sekar et al., 2022). In this scenario, age, gender, genetic factors, and lifestyle factors have been significant contributors to the increasing number of patients, with the risk in men being generally at a younger age when compared to women, whose risk is substantially increased after menopause (El-Shafiey et al., 2022; Eltrass et al., 2021). Individuals aged above 65 suffer extensively. Since so many people worldwide suffer from heart disease, its early detection is important in managing the illness well. Identifying heart disease at its onset allows health providers to take preventive measures, initiate treatment plans, and perhaps save lives (Eltrass et al., 2021). Current deep-learning approaches in identifying cardiac diseases like CNN are usually affected by dataset skewness, which reduces accuracy for less frequent diseases (Fradi et al., 2022). CNN-LSTM systems enhance temporal modelling but suffer from noisy or low-quality labels. Reinforcement learning can alleviate the data imbalance issue but it consumes a lot of computational resources and the choice of reward functions is rather sensitive (Hossain et al., 2023; Almulihi et al., 2022). These methods require further improvement in terms of noise management and computational overhead. The suggested work is aimed at eradicating these drawbacks for improved and accurate prediction of heart diseases.

The advent of deep learning and federated learning heralds a promising solution in advancing improvements in the diagnosis and treatment of HD. Deep learning, which is a subset of AI shows remarkably high success in recognizing patterns in medical complex data, such as electrocardiograms or images with far greater accuracy than conventional techniques (Nancy et al., 2022; Yaqoob et al., 2022). In addition, federated learning can enable collaborative, privacy-preserving model training across distributed healthcare systems, so sensitive patient data remains secure and is used for the benefit of collective insights. By combining deep learning’s diagnostics with federated learning’s decentralized approach, the health industry can develop more accurate, personalized, and efficient heart disease detection systems that can increase diagnosis accuracy and improve patient care outcomes (Otoum et al., 2024; Li et al., 2023; Al-Issa and Alqudah, 2022). A novel approach is proposed by combining DL and FL to overcome the existing drawbacks of current techniques, such as data imbalance, privacy concerns, and computational inefficiencies, resulting in a more robust and efficient heart disease detection system.

The major contribution of this research work include:

• The integration of federated learning ensures secure, decentralized model training across multiple devices while preserving data privacy, making the approach scalable and efficient for real-world healthcare applications.
• The use of diverse multimodal datasets, including cardiac images, ECG signals, patient records, and nutrition data, allows for a comprehensive analysis of cardiac diseases, improving the model’s ability to make accurate predictions.
• The adopt an attention-based feature fusion model for effectively integrating and prioritizing critical information from various data sources, enhancing the overall diagnostic performance and reducing redundancy
• To implement SGD-DNN Model Training for improving the model’s accuracy and adaptability to node-specific data, refining predictions for diverse patient profiles.
• To suggest lifestyle recommendation by DRL for patients identified as positive cases, promoting better health outcomes through targeted, individualized care strategies

### 1.1 The organization of the article

Section 2 represents the literature review based on cardiac disease detection, followed up in section 3 with the stated methodology. Section 4 contains the result and discussion and finally the work is concluded by conclusion and future scope.

## 2 Related works

In 2021, Khan et al., 2021 suggested a generalized approach for processing of ECG images of all types for identifying cardiac disorders. Cardiovascular diseases are detected using a Deep Neural Network architecture called Single Shot Detection (SSD) MobileNet v2. The proposed system focuses on identifying four major cardiac abnormalities like myocardial infarction, abnormal heart beat, history of previous MI and normal class.

In 2021, Mehmood et al. (2021) introduced a method known as CardioHelp that estimates the likelihood of CVD in a patient using the CNN deep learning (DL) algorithm. The suggested method is related to the temporal data modeling by CNN for the prediction of the heart failure (HF) at its initial stage.

In 2022, Wang et al. (2022) introduced a new wireless ECG patch alongside a deep learning system using CNN and LSTM. To overcome the limitations in identifying different types of heartbeat, it presents a semi-supervised method to use the confidence-level-based training for the badly labeled data samples to increase the classification accuracy.

In 2023, Ali et al. (2023) presented LU-Net, a deep encoder-decoder architecture for removing noise from heart sound signals recorded using digital stethoscopes in noisy conditions. When fine-tuned on a benchmark PCG dataset, LU-Net is capable of attenuating ambient and physiological noises and demonstrates higher SNR and classification accuracy than the U-Net and FCN models. This approach substantially improved the diagnostic performance in the low resource environment and in the presence of noise.

In 2023, Matten et al. (Yaqoob et al., 2023) presented a hybrid model for HD prediction. At the client end it uses Modified Artificial Bee Colony Optimization with Support Vector Machine (MABC-SVM) for the purposes of feature selection as well as classification. For privacy at the server end, it employs what is known as Federated Matched Averaging. The proposed framework is tested on a combined cardiovascular disease dataset and shows better prediction of the model, less classification error, and fewer training iterations compared to conventional federated learning (FL) methods.

In 2023, Amir et al. (Khan et al., 2023) The research puts forward an Asynchronous Federated DL Approach for Cardiac Prediction (AFLCP) to combine a HD dataset with deep neural networks (DNNs) through a FL approach. The method asynchronously updates DNN parameters and uses a temporally weighted average update to enhance the accuracy and convergence of the central model. The performance of AFLCP is tested on two datasets using different DNN structures and is shown to outperform baseline approaches based on the required communication cost and model quality.

In 2022, Khozeimeh et al. (2022) proposed an approach for identification of CAD using CMR images. This is done through the integration of DL with random forest through the feature extraction of convolutional neural networks (CNNs). The method is named RF-CNN-F, which transforms image data into numeric features for classification; the accuracy is 99.18% while the accuracy of the CNN is 93.92% on average. This approach is intended to be generalizable across any image dataset, and is demonstrated here to improve CAD detection.

In 2023, Sudha and Kumar (2023) presented CNNs in conjunction with LSTM networks for the prediction of HD. This method is designed to obtain better results compared to classical machine learning (ML) algorithms. The CNN and LSTM model was trained on a HD dataset and obtained an accuracy of 89% and was cross-checked by using k-fold cross-validation. The findings showed that this combined approach is superior to different ML techniques such as SVM, Naïve Bayes, Decision Tree.

In 2024, Gayathri et al. (2024) suggested an approach for enhancing the prediction of HD through integrating data augmentation into reinforcement learning (RL). This fusion methodology improves the predictive models by adding data and uses RL for decision making in sequences, with a success rate of 94%. The approach helped to solve the problems of working with high-dimensional cardiac data and increase the effectiveness of patient treatment and diagnosis.

In 2024, Mirzaee et al. (Kasmaee et al., 2024) suggested an enhanced deep model known as ELRL-MD that incorporates ensemble learning and RL for diagnosis of myocarditis from cardiac magnetic resonance (CMR) images. The model incorporates pre-training by the artificial bee colony (ABC) algorithm to improve learning and a set of CNNs to extract and combine features for diagnosis. It employs RL to solve the problem of data imbalance in the dataset as diagnosis is formulated as a decision-making problem. The Table 1 denotes the comparison of the existing techniques of cardiac disease detection.

**TABLE 1:** Comparison of the existing techniques of cardiac disease detection.

Authors, Year	Techniques	Databases	Advantages	Limitations	Outcomes
Khan et al. (2021)	SSD MobileNet v2, Deep Neural Networks	11,148 standard 12-lead ECG images	High accuracy, domain expert-annotated data	Limited to 4 major cardiac abnormalities	Accuracy: 98%
Mehmood et al. (2021)	CNN	Heart disease dataset	High accuracy, good performance for temporal data modeling	No direct comparison with advanced deep learning methods	Accuracy: 97%
Wang et al. (2022)	CNN, LSTM, Semi-supervised method	Newly obtained ECG dataset	Improved accuracy with confidence-level-based training	Existing models fail on new dataset	Accuracy: 90.2%
Ali et al. (2023)	Deep Encoder-Decoder-based Denoising (LU-Net)	Benchmark PCG dataset, PASCAL heart sound dataset	Effective noise suppression, improved SNR	Requires clean heart sound recordings	RMSE: 0.097
Yaqoob et al. (2023)	MABC-SVM, Federated Learning	Combined cardiovascular disease dataset	Privacy preservation, reduced training latency	Higher complexity due to federated learning	Accuracy: 93.8%
Khan et al. (2023)	Asynchronous Federated Deep Learning, DNNs	Heart disease dataset	Improved accuracy and convergence, reduced communication cost	High complexity, federated system	Accuracy: 87.8% Precision: 87.7%
Khozeimeh et al. (2022)	Random Forest, CNN Features	CMR dataset	High accuracy, non-invasive	Needs numeric conversion for CNN features	Accuracy: 99.18%
Sudha and Kumar (2023)	CNN, LSTM	Heart disease dataset	High accuracy, hybrid system	Comparison limited to basic machine learning models	Accuracy: 89%
Gayathri et al. (2024)	Data Augmentation, Reinforcement Learning	Cardiac disease dataset	Improved predictive accuracy, better handling of complex data	Complex method, requires more computational resources	Accuracy: 94%
Kasmaee et al. (2024)	Ensemble Learning, Reinforcement Learning (RL), CNN	Z-Alizadeh Sani myocarditis CMR dataset	High efficacy, addresses dataset imbalance	Dependent on pre-training and RL setup	F-measure: 88.2%, Geometric mean: 90.6%

In 2019, Javeed et al. (2019) developed an algorithm for the Random Search Algorithm (RSA)-based feature selection coupled with optimized Random Forest model to improve heart disease detection accuracy to about 93.33% with lesser features. Another example of nature-inspired optimization by training Multilayer Perceptron (MLP) with Multi-verse Optimizer (MVO) to showcase what such techniques can do has already been illustrated by Jalali et al. (2019), also in 2019. Dhanka and Maini (2021), in 2021, and Latha and Jeeva (2019) Latha and Jeeva in 2019 used Random Forest and ensemble methods, respectively, but to lower accuracies than other approaches. HAQ et al. (2019) and Li et al. (2020) focused on various machine learning and deep-learning classifiers, respectively, in the year 2019 and 2020, with the outcome achieving high accuracy using BPNN and SVM-based approaches. All these studies generally share the major demerit of being based on single datasets, which do not generalize into the wider picture and ignore data privacy, which is critical in healthcare. As much as they are most likely to be based regarding the IoT and cloud architectures for health monitoring, Kumar and Gandhi (2018), and Ahmed et al. (2018) emphasized in 2018 without really sleeping at the address of personalization. The reasons motivating our federated learning-based multimodal development include lack of data privacy and dependence on only single datasets, making better use of the capability that multimodal data integration brings. It is based on federated learning; hence, data is decentralized for better privacy, while the attention-based feature fusion combines in the best possible way in the image, ECG signals, patient records, and nutrition vis-a-vis disease detection.

For early detection of severe left ventricle hypertrophy (SLVH) and dilated left ventricle (DLV), Wang et al. (2024) proposed a model of multimodal fusion driven by VAE, which fuses structured data collected in CXR with chest X-ray images. Although this model showed superior accuracy to existing methods, it was only possible to validate it using datasets from one institutional source, thus putting it into question generalizability. In 2024, Yousuf et al. (2024) presented a 2D-CNN methodology relying on Gramian angular field (GAF) conversion to detect inferior myocardial infarction (MI) from lead II ECG signals. While its classification accuracy was great, it could not give a thorough diagnosis of the heart due to its dependency on one ECG lead. In 2024, Pu et al. (2024) developed a hybrid neural network for detecting normal cardiac cycles in fetal ultrasound videos. His work improved the recognition of anatomical structures but is limited as it requires high-quality fetal ultrasound data, making it impractical for use in common real-world situations.

Furthermore, in 2024, Vinay et al. (2024) adopted a Bi-LSTM-based GAN for analyzing heart sound signals meant for CVD detection. They synthesized pseudo-real data that were of superb quality, but their reliance on these old GAN systems may usually introduce unstable training. Amiri et al. (2025) proposed an optimized deep active learning (ODAL) framework on arrhythmia detection that improved sensitivity and specificity over the former classifiers in 2025. They did not explain how to relate and integrate multimodal data sources for a more holistic diagnosis. The above methods have their setbacks mostly in limited dataset dependency, suboptimal fusion of multimodal data, and very high false-positive or negative rates. To cater for these setbacks, an integrated framework will collect and fuse multimodal health data including cardiac images, ECG signals, patient records, and nutrition data, so as to improve the accuracy of diagnosis. An attention-based feature fusion model will be used to extract relevant cross-modal features such that improved benefits are derived from the information. It brings in federated learning to promote data privacy yet maintains scalability across distributed sources. For more predictive accuracy, a deep neural SGD-DNN trained locally model will further boost this. Unlike any other previous works, this frame mind does not aim at accurate disease prediction only but also offers lifestyle recommendations for positive cases, thus improving preventive care. Multiple metrics of performance evaluation confirmed the effectiveness of the model, achieving very high accuracy rates: 97.76% in Database 1, 98.43% in Database 2, and 99.12% in Database 3.

### 2.1 Problem statement

## 3 Federated learning with cardioNet + -based cardio-disease detection and lifestyle recommendation

**FIGURE 1:**
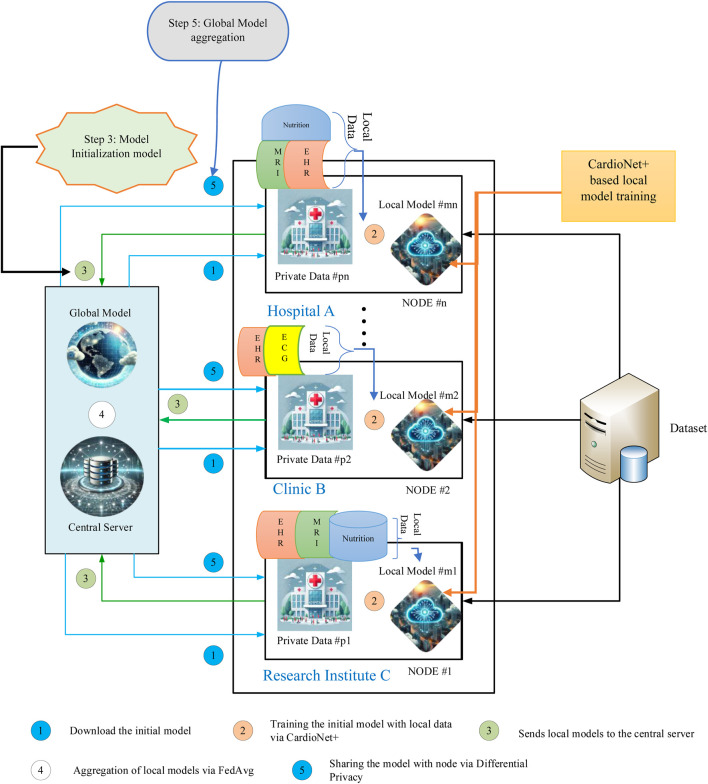
Proposed methodology.

FL is a ML framework in which multiple nodes collaboratively contribute to the training of a more robust and efficient global model for heart disease detection while maintaining decentralized data storage. Figure 1 illustrates an overview of the deployed FL architecture with local training *via* cardioNet + framework. In this study, publicly available datasets containing heart disease-related medical data, such as cardiac images (MRI), ECGs and patient records (EHRs), Meta-data (nutrition data) were used to simulate a network of collaborative nodes that collectively enhance the global model’s diagnostic performance.

The proposed methodology is presented in the following five fundamental stages, as depicted in Figure 1, to train and develop a reliable and secure global heart disease detection model while maintaining data confidentiality.

### 3.1 Phase 1: model initialization

In the first stage, a preliminary heart disease detection model is established on a central server. This model is then disseminated to several local nodes (for instance hospitals or clinics, or any healthcare facilities) in the network, each of which has its local data for training. This makes sure that all the nodes start from the same level or from the same point.

### 3.2 Phase 2: personalized training

Every local node is trained on the initial model and its local dataset which can be heart-related data, such as ECG, images, patient records and Nutrition Data. The use of this localized approach ensures that the training process includes regional patterns and variation while avoiding the use of privileged information.

**FIGURE 2:**
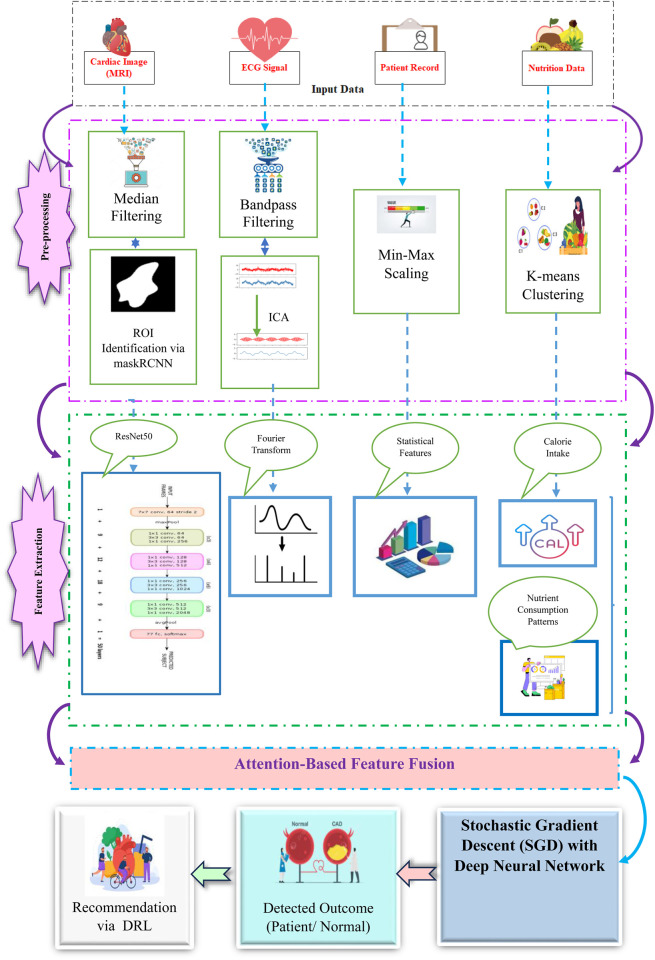
Framework of CardioNet+.

The obtained raw data, such as cardiac images, ECG signals, patient records and nutrition data, is pre-processed to improve data quality and data coherence. Subsequent to data preprocessing, the data is used to diagnose and identify the presence of cardiac abnormalities. The Figure 2 represents the proposed framework of CardioNet+.

#### 3.2.1 Data preprocessing:

##### 3.2.1.1 Cardiac image preprocessing and segmentation

**FIGURE 3:**
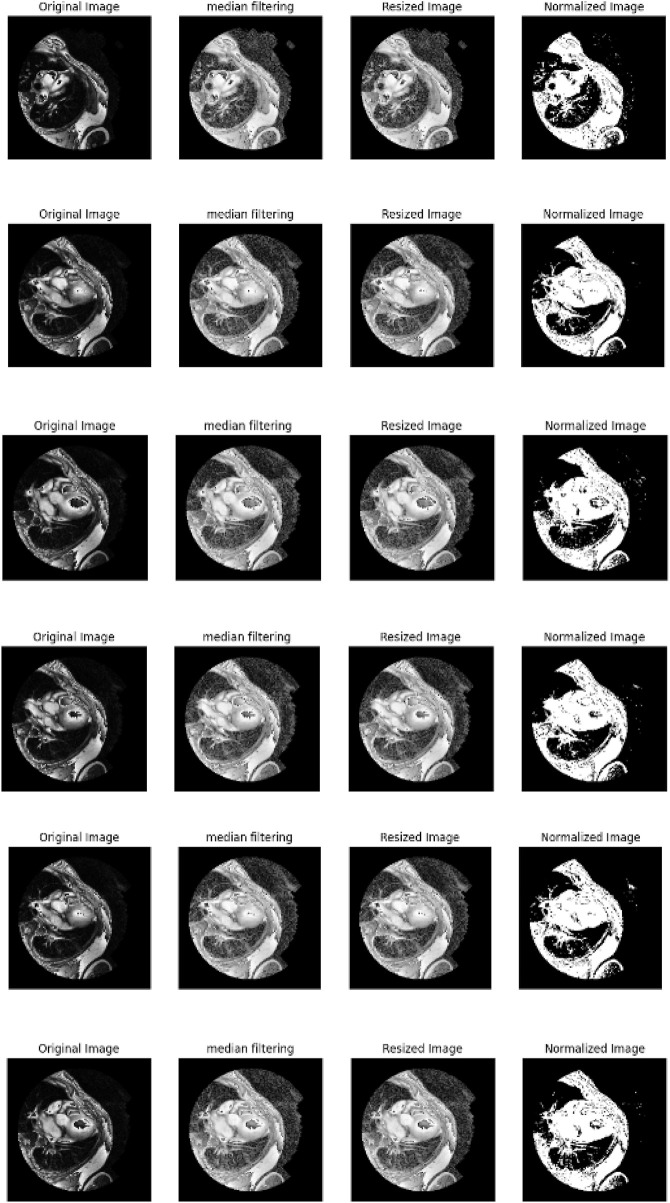
Pre-procced cardiac images.

•Denoising using median filtering: Median filtering is used to filter out noise while at the same time preserving edges in images of the cardiac patient. For a pixel $I\left(x,y\right)$ in the image, the denoised value is given by Equation 1. The pre-procced cardiac images are represented in Figure 3.

$$I^{\prime }\left(x,y\right)=median\left\{I\left(i,j\right):\left(i,j\right)\in N\left(x,y\right)\right\}$$ (1)

where $N\left(x,y\right)$ represents the neighborhood of pixel $\left(x,y\right)$

• Segmentation using Mask R-CNN: Mask R-CNN divides the cardiac image into ROIs to analyze the image further. The segmentation process involves a two-step optimization: Bounding box regression and mask prediction. The loss function is given by Equation 2:

$$L=L_{cls}+L_{bbox}+L_{mask}$$ (2)

where $L_{cls}$ ​ is the classification loss, $L_{bbox}$ ​ is the bounding box regression loss, and $L_{mask}$ ​ is the binary mask loss.

##### 3.2.1.2 ECG pre-processing

• Bandpass Filters: For the ECG signals noise and baseline drift are removed through filtering. The bandpass filter can be defined as Equation 3:

$$H\left(f\right)=\left\{\begin{array}{cc} 1 & f_{1}\leq f\leq f_{2}\\ 0 & otherwise \end{array}\right.$$ (3)

where $f_{1}$ ​ and $f_{2}$ are the lower and upper cutoff frequencies, respectively.

• Independent Component Analysis (ICA): ICA splits mixed signals into independent sources, which is widely applied to artifact elimination in ECG. In the case of a mixed signal X , ICA assumes it to be as Equation 4.

X=AS (4)

where A is the mixing matrix, and S represents the independent components. ICA estimates S by maximizing statistical independence.

##### 3.2.1.3 Patient record preprocessing

• Min-Max scaling: Patient records are normalized to bring all features into a consistent range [0,1]. The mathematical expression for Min-Max scaling is shown in Equation 5:

$$H^{\prime }=\frac{H-H_{\min}}{H_{\max}-H_{\min}}$$ (5)

where H is the original value, ​ $H_{\min}$ is the minimum value, and ​ $H_{\max}$ is the maximum value of the feature. This ensures that all features contribute equally to the model.

##### 3.2.1.4 Nutrition data preprocessing

• K-means clustering for grouping patients: These patients are grouped by means of dietary patterns using K-means, in a way that optimizes the variance within each cluster. This can be given mathematically as per Equation 6.

$$J=\sum_{i=1}^{k}\sum_{x\in C_{i}}∥x=\mu_{i}{∥}^{2}$$ (6)

where $C_{i}$ ​ represents the i -th cluster, $\mu_{i}$ is the centroid of cluster $C_{i}$ , and $∥x=\mu_{i}{∥}^{2}$ is the Euclidean distance between a data point x and the cluster centroid. Patients can be grouped according to their nutritional profile and the specific diets that they consume and thus, specific recommendations made to augment heart disease management.

#### 3.2.2 Feature extraction

##### 3.2.2.1 Cardia image features

###### 3.2.2.1.1 ResNet50 for extracting spatial features

ResNet50 has shown hierarchical feature extraction capabilities and was first trained on the large ImageNet dataset. ResNet50 is a deep CNN architecture that is used in extracting spatial features from the cardiac images for heart disease classification. The ResNet50 model does not have the vanishing gradient problem and deepens the network by using skip connections through residual learning. During this process, multiple convolutional layers, pooling, and fully connected layers are included in the process of ResNet50 network operations. The extracted feature vector is obtained at the last layer to represent the core features of a cardiac image, and the produced output vector in that network could lead to classification while further processing provides higher-level spatial features. The initial layers of ResNet50 extract low-level spatial features (edges, textures) common to various domains, including medical imaging. Because some rich spatial features are already in its awareness, fine-tuning can be accomplished fairly easily and computationally efficiently with fewer epochs and less training data.

$$F=ResNet50\left(I\right)$$

Here, F is the feature vector, and I is the input cardiac image.

##### 3.2.2.2 ECG signal features

###### 3.2.2.2.1 Fourier transform to capture dominant frequencies

In this work, the Fast Fourier Transform (FFT) is applied to examine the frequency domain of the ECG signal. It converts the ECG signal obtained in the time domain into its frequency domain so as to capture dominant frequencies related to the heart disease like arrhythmias.

The Fourier Transform of a time-domain signal $x\left(t\right)$ is given by Equation 7:

$$X\left(f\right)=\int_{-\infty }^{\infty }x\left(t\right)e^{-j2\pi ft}dt$$ (7)

where $X\left(f\right)$ is the frequency spectrum of the signal, f is the frequency and $x\left(t\right)$ is the ECG signal in the time domain. Moreover, by studying the frequency components, it is possible to distinguish certain patterns that are characteristic of heart diseases.

##### 3.2.2.3 Patient data features

###### 3.2.2.3.1 Statistical features (mean, variance, and standard deviation)

Various statistical characteristics are important for assessing the general state of health of a patient. These features describe the mean and spread of the data and can be utilized to detect the presence of HD.

For a given feature vector $X=\left\{x_{1},x_{2},\ldots ,x_{n}\right\}$ which contains data of the patient (e.g., blood pressure, cholesterol levels) the following statistical features are computed:

• *Mean:* The mean gives the general value of the feature, which is defined by Equation 8.

$$\mu =\frac{1}{n}\sum_{i=1}^{n}x_{i}$$ (8)

• *Variance:* Variance is a measure of how far apart data points lie from the mean value, which is given by Equation 9.

$$\sigma^{2}=\frac{1}{n}\sum_{i=1}^{n}{\left(x_{i}-\mu \right)}^{2}$$ (9)

• *Standard Deviation:* Standard deviation means the extent of spread of the data or the variability of the data as provided in Equation 10.

$$\sigma =\sqrt{\sigma^{2}}$$ (10)

These statistical features are useful in establishing the patterns of the data that are characteristic of heart disease because they show the amount of spread or agreement of vital health factors.

##### 3.2.2.4 Nutrition data features

###### 3.2.2.4.1 Feature engineering (calorie intake, nutrient consumption patterns)

Information regarding nutrition can be useful in a patient’s medical profile especially as a predictor of heart disease factors like cholesterol and blood pressure levels. Feature engineering in this case is the process of extracting meaningful nutrition related features from raw dietary data.

###### 3.2.2.4.2 Calorie intake

The total calories of a patient can be determined by adding the calories taken per meal or per day. This can be an important feature for understanding the patient’s eating behaviors and possible danger of cardiovascular disease. The calorie intake can be computed utilizing Equation 11.

$$TotalCalorieIntake=\sum_{i=1}^{n}{calories}_{i}$$ (11)

###### 3.2.2.4.3 Nutrient consumption patterns

Thus, if there is a possibility to analyze nutrient consumption, meaningful features can be derived from fats, carbohydrates, and proteins intake. For example, the degree of saturation of fats to total fats or macronutrient distribution ratio can be used to evaluate the quality of the patient’s diet. These patterns can be calculated as Equation 12, 13:

$$FattoTotalFatRatio=\frac{SaturatedFat}{Total\;Fat}$$ (12)

$$ProteintoCarbohydrateRatio=\frac{ProteinIntake}{Carbohydrate\;Intake}$$ (13)

These features give a quantitative description of patient’s nutrition and can be used for better prediction and classification of heart diseases risks according to patients’ diet.

#### 3.2.3 Attention based feature fusion

The features extracted from cardiac images, ECG signals, patient records, and nutrition data are seamlessly integrated using an attention-based feature fusion mechanism. This makes it possible to capture only the most informative features from each modality and improve the final decision-making by combining them.

All modalities’ features are concatenated together to form the unified representation vector z . Attention Mechanism assigned weights to such features to allow the model to selectively emphasize important information.

$$z=Concat\left(Cardiac,ECG,PatientRecord,NutritionFeatures\right)$$

##### 3.2.3.1 Incorporating attention

For increasing z , an Attention Score is calculated for each modality to facilitate modulation and weighted combination. For a feature set $F_{i}$ the attention weight $\alpha_{i}$ is defined as Equation 14:

$$\alpha_{i}=\frac{\exp\;\left(Score\left(F_{i}\right)\right)}{\sum_{j=1}^{n}\exp\left(Score\;\left(F_{j}\right)\right)}$$ (14)

$$Score\left(F_{i}\right)=W_{a}^{T}F_{i}+b_{a}$$

Which computes the relevance of modality i with trainable parameters $W_{a}$ and $b_{a}$ ​. The $\alpha_{i}$ denotes the normalized attention weight for modality i . The final fusion vector $z_{fused}$ ​ is given by Equation 15:

$$z_{fused}=\sum_{i=1}^{n}\alpha_{i}F_{i}$$ (15)

**FIGURE 4:**
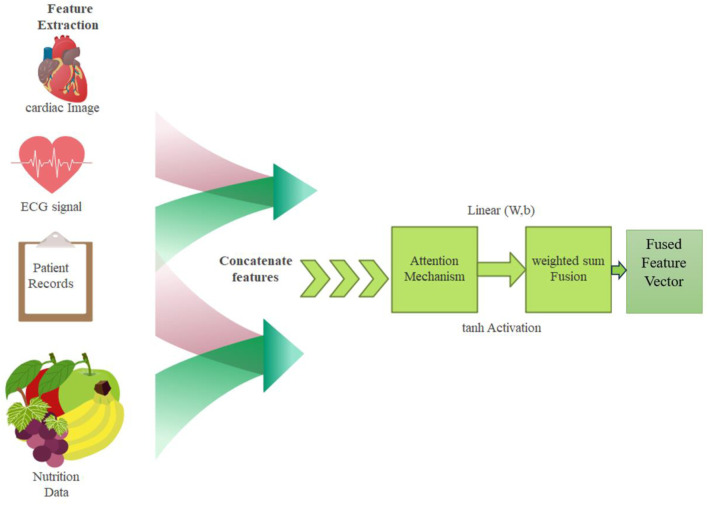
Attention-based feature fusion.

Multimodal models improved through attention-based fusion dynamically weights the critical features for improving attention to relevant information. It has boosted interpretability through identification of influential modalities and robustness through effective integration of diverse data for more accurate and reliable detections. The attention-based feature fusion is explained in Figure 4.

#### 3.2.4 Model training - stochastic gradient descent (SGD) with deep neural network for local model fine-tuning on the node-side

The training of a model in federated learning heavily relies on efficient local fine-tuning to fine-tune the global model towards each node’s specific data distribution. Stochastic Gradient Descent is an optimization algorithm that has to be applied at this phase; it proceeds with iteratively adjusting the parameters of the model using the gradient of the loss function calculated from mini-batches of the local data. On the node-side, a Deep Neural Network (DNN) is used as the model for learning of complex patterns and relationships from multimodal data including cardiac images, ECG signals, patient records, and nutrition data.

In the federated learning setup, each node employs a Deep Neural Network (DNN) to fine-tune the global model locally using its own dataset. The optimization process is carried out using Stochastic Gradient Descent (SGD), which iteratively adjusts the network parameters to minimize the loss function. The architecture of the DNN is crucial for effectively capturing complex patterns from multimodal data, including cardiac images, ECG signals, patient records, and nutrition data. The proposed DNN architecture consists of multiple layers, specifically designed to ensure optimal feature extraction, robustness, and generalization across different data distributions at each node. The DNN is composed of five hidden layers flanked by an input layer on one side and an output layer on the other. The number of neurons varies from layer to layer to extract hierarchical features at different levels of abstraction. The complete architecture can be described as follows:

##### 3.2.4.1 Input layer

The input layer receives multimodal data with possible image-based features (like MRI and ECG signals) and structured numerical data (like patient records and clinical variables). The input dimensions depend on feature engineering and preprocessing steps.

##### 3.2.4.2 Hidden layers:

a. First Hidden Layer: Fully connected layer with 512 neurons, activated using Rectified Linear Unit (ReLU) function capturing high-dimensional abstract features from the input data
b. Second Hidden Layer: Fully connected layer with 256 neurons, again activated through ReLU, incorporated to make the representation of the feature nonlinear and thus potentially richer.
c. Third hidden layer: Fully connected layer with 128 neurons, but here batch normalization is added for stabilization of the learning process and improved generalization.
d. Fourth hidden layer: Fully connected layer with 64 neurons where dropout (rate = 0.3) is used to prevent overfitting by randomly turning off neurons during training.
e. Fifth Hidden Layer: Fully connected with 32 neurons and is thereby considered a bottleneck layer directed at the best features sorted out before the last classification.

##### 3.2.4.3 Output layer

The last layer of the architecture consists of a fully-connected layer that applies softmax to obtain flush classification, in which the number of output neurons equals the classes within the target variable (e.g., heart disease classes for classification).

##### 3.2.4.4 SGD optimization and gradient computation

The SGD algorithm aims at adjusting the DNN parameters to make the loss function obtain a minimum value. For each mini-batch $x_{i}$ and its corresponding label $y_{i}$ ​, the parameter updates are computed as Equation 16:

$$\theta \leftarrow \theta -\eta \nabla_{\theta }L\left(f\left(x_{i},\theta \right),y_{i}\right)$$ (16)

Where, θ represents the model parameter, η indicates the learning rate, $\nabla_{\theta }L$ for the gradient of the loss function with respect to θ , the loss function is denoted by L and $f\left(x_{i},\theta \right)$ signifies the model detection for input $x_{i}$ .

##### 3.2.4.5 Gradient computation for mini-batches

To enhance computational speed, gradients are computed over mini-batches B rather than over individual samples as Equation 17:

$$\nabla_{\theta }L\left(B;\theta \right)=\frac{1}{\left|B\right|}\sum_{i\in B}\nabla_{\theta }L\left(f\left(x_{i},\theta \right),y_{i}\right)$$ (17)

##### 3.2.4.6 Regularization for generalization

To avoid overfitting and increase model’s ability to generalize, L2 is incorporated into the loss function as Equation 18:

$$L_{reg}=L+\frac{\lambda }{2}∥\theta^{2}∥$$ (18)

Here, λ denotes the regularization coefficient, $∥\theta^{2}∥$ signifies the squared norm of the model parameters. The updated parameter rule with regularization becomes as Equation 19.

$$\theta \leftarrow \theta -\eta \left(\nabla_{\theta }L\left(B;\theta \right)+\lambda \theta \right)$$ (19)

##### 3.2.4.7 Momentum for accelerated convergence

To add more robustness and to increase the rate of convergence, momentum is integrated into SGD, which is defined as Equation 20.

$$v^{t+1}=\gamma v^{t}+(\nabla_{\theta }L\left(B;\theta \right)$$ (20)

$$\theta^{t+1}=\theta^{t}-v^{t+1}$$

Here, $v^{t}$ denotes the velocity term and γ for the momentum factor.

Through SGD, the local model continues to adjust its parameters and thus learn patterns within the node’s data. This localized adaptation is important for enhancing the detection accuracy especially in environments where the data characteristics differ from one node to the other.

##### 3.2.4.8 Federated learning integration

In a synchronized manner, each local node trains the model based on its own dataset and updates the parameters. The updated local models are then aggregated globally using techniques such as Federated Averaging (FedAvg) to create a more generalized global model. Through federated learning, patient-sensitive data is guaranteed to stay on local devices, thereby enhancing privacy, while also ensuring that the classification performance remains at a high level. In the present localized adaptation process, the SGD-DNN model can effectively extract complex patterns from each node’s non-IID dataset, hence improving detection capabilities, mostly in non-IID scenarios where such characteristics in data vary across nodes. Together, deep learning and SGD-based optimization grant strong adaptability and scalability to federated learning systems. The parameter and their values of SGD-DNN model is shown in Table 2.

**TABLE 2:** SGD-DNN parameters and values.

Parameter	Symbol	Value/Description
Weight Initialization	-	Xavier/He Normal initialization
Regularization Coefficient	λ	0.001 (L2 regularization)
Optimization Algorithm	-	Stochastic Gradient Descent (SGD) with Momentum
Number of Hidden Layers	-	5
Neurons in Hidden Layer 5	-	32
Neurons in Hidden Layer 4	-	64
Neurons in Hidden Layer 3	-	128
Neurons in Hidden Layer 2	-	256
Neurons in Hidden Layer 1	-	512
Momentum Factor	γ	0.9 (for momentum-based SGD)
Loss Function	L	Cross-Entropy Loss
Learning Rate	η	0.01 (adjustable based on convergence)
Global Model Aggregation	-	Federated Averaging (FedAvg)
Dropout Rate	-	0.3 (for regularization in hidden layers)
Batch Normalization	-	Applied to Hidden Layer 3
Activation Function	-	ReLU (for hidden layers), Softmax (for output layer)

#### 3.2.5 Analysis on SGD with momentum over AdamW

Optimization is of critical importance for all the working aspects of deep learning models, such as convergence speed, stability, and generalization capability. AdamW and its variants, owing to their adaptive learning rates and weight decay enhancements, have emerged as firm favorites over time. However, it is the Stochastic Gradient Descent (SGD) momentum variant that is one of the most exploited and successfully used in various machine learning tasks, including our proposed model. To validate our whole argument, we empirically performed an extensive performance comparison between SGD with momentum and AdamW concerning several evaluation metrics, such as accuracy, precision, recall, F1-score, and speed of convergence. The results acquired are manifested in Table 3.

**TABLE 3:** Performance comparison between SGD with momentum and AdamW.

Optimizer	Accuracy	Precision	Recall	F1-Score	Training Time (Epochs)
SGD with Momentum	99.50%	99.60%	99.50%	99.50%	45 epochs
AdamW	99.30%	99.40%	99.30%	99.30%	52 epochs

SGD with Momentum surpasses AdamW in the accuracy figures, with a 99.50% recorded accuracy against the former’s 99.30%, indicating the superior generalizing power of the model. The F1-score has a similar trajectory in both models (99.50% versus 99.30%), indicating that SGD is more balanced in precision and recall. In fewer epochs, SGD has converged, 45 as opposed to 52 for AdamW, establishing SGD as computationally much more economical. Such phenomena indicate that SGD with momentum is a most promising approach to learning. The efficacy of these results was evaluated statistically using a paired t-test and Wilcoxon Signed-Rank Test. The results acquired are manifested in Table 4 and Table 5, respectively.

**TABLE 4:** Paired t-test Results.

Comparison	t-statistic	p-value	Significance
SGD vs. AdamW	4.85	p < 0.001	Significant

**TABLE 5:** Wilcoxon signed-rank test results.

Comparison	Z-score	p-value	Significance
SGD vs. AdamW	5.21	p < 0.001	Significant

The very small statistically significant p-values (<0.001) confirmed the statistical differences into SGD momentum outperforming AdamW. These findings made it possible to conclude that adaptive optimizing methods are less suitable for the model and rather had the following qualities that made it preferable:

• Improved generalization: Lower chance of overfitting compared with adaptive optimizers like AdamW.
• Faster convergence: requires fewer epochs for optimal performance.
• More stable updates: no sudden jumps, oscillation of weights.

For future researchers, hybrid optimizers taking advantage of both SGD and AdamW, e.g., Lookahead-SGD or AdaBelief, may be further avenues for increasing performance. SGD with momentum is the superior optimizer theoretically and empirically based on our analysis. The results obtained from performance metrics, statistical significance tests, and loss convergence curves serve as strong justifications for the choice made. Advanced optimizers could be researched in the future to improve efficiency further.

#### 3.2.6 Nutrition recommendation via DRL

**FIGURE 5:**
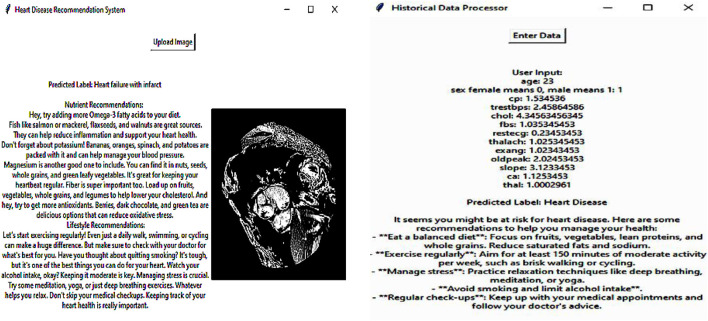
Nutrition recommended results.

The system collects the outcome of the detected heart disease through the developed DL model and then recommends appropriate nutrition based on the detected results. The patient’s response to the previous dietary suggestions includes changes in health parameters or self-perceived health status. The DRL framework is designed to find out the appropriate nutrition based on the detected outcomes. The DRL agent represents the recommendation system to choose the diet. It interacts with the environment, particularly the patient’s health status and response, to deciding on nutrition plans. This process involves the application of neural networks to predict the value of different diet actions. The DRL system is intended to reflect on the patient’s response and the patient’s health condition. That makes the recommendations personally beneficial in the long run since they are tailored to the individual. With the help of DRL, the nutrition recommendation system offers patient-specific recommendations, that are dynamic and evolving as the patient’s health condition changes, which is useful in the treatment of HD and the patient’s overall wellbeing. The nutrition-recommended results of the sample are presented in Figure 5.

The Deep Q-Network (DQN) is a value-based Deep Reinforcement Learning (DRL) algorithm suitable for personalized recommendation systems, which forms the cornerstone of this proposal. It maximizes cumulative rewards derived from user interactions to learn an optimal recommendation policy. The DQN differs from traditional recommendation methods which are static in nature and does adapt to changing user preferences across time. A system using Deep Q-Networks (DQN) for optimizing health outcomes will improve on past patients' feedback and change consequences for their updated diets. The DQN-based system for cardiac disease recommendation operates in a reinforcement learning framework where the agent (AI model) suggests dietary plans, and the patient’s response serves as feedback to refine future recommendations. The system consists of the following components:

##### 3.2.6.1 Components

• State Representation: The patient’s current health profile, including biometric parameters (e.g., blood pressure, cholesterol, BMI), dietary habits, and self-reported wellbeing.
• Action Space: A set of possible dietary recommendations (e.g., increase fiber intake, reduce sodium, include omega-3 fatty acids).
• Reward Function: Defined based on improvements in patient health indicators and adherence to recommendations.
• Q-Network: A deep neural network that learns to predict the **expected future reward** for each dietary action.
• Experience Replay: A buffer that stores previous interactions to stabilize learning and prevent overfitting.

##### 3.2.6.2 Patient response as feedback for learning

After receiving a dietary recommendation, the patient’s response is monitored through:

• Changes in health parameters: Reduction in blood pressure, cholesterol levels, or BMI after following a suggested diet.
• Self-perceived health improvements: Patients may report increased energy, better sleep, or reduced symptoms like chest pain.
• Adherence score: Captures how well the patient follows the recommendations, influencing future suggestions.

This feedback is used to update the **Q-values** in the DQN, ensuring that recommendations evolve based on individual patient progress.

##### 3.2.6.3 Transformer integration for sequential learning

Since dietary habits and health trends evolve over time, a **transformer-based module** (LSTM-based attention) can be integrated with the DQN. This enables:

• Capturing long-term trends in patient responses.
• Improving sequential decision-making for dietary adjustments.
• Personalizing recommendations based on past dietary adherence.

##### 3.2.6.4 Training and evaluation

The model is trained using **historical patient data** and validated using real-world feedback. The evaluation metrics include:

• Health improvement score: Measures the impact of dietary changes on key health parameters.
• Recommendation adherence: Tracks how consistently patients follow AI-generated dietary plans.
• Patient satisfaction index: Evaluates user-reported wellbeing after dietary modifications.

The data for cardiac recommendation has been generated from Kaggle Diet Recommendations Dataset (https://www.kaggle.com/datasets/ziya07/diet-recommendations-dataset), Personalized Medical Diet Recommendations Dataset (https://www.kaggle.com/datasets/ziya07/personalized-medical-diet-recommendations-dataset), respectively.

### 3.3 Phase 3: secure parameter sharing via differential privacy

Local training is performed by each node, and at the end of the process, each of them sends updates of the model’s parameters. These updates are securely transferred to the central server while at the same time the raw patient data is kept within the local nodes. During this phase, the highest level of encryption is used to ensure that the information is not distorted or intercepted.

Differential Privacy does this by introducing carefully calibrated noise into the updates on the parameters before they are shared. Let ΔW denote the raw model parameter updates generated in local training. To protect these updates, noise sampled from a Gaussian distribution $N\left(0,\sigma^{2}\right)$ is added, as Equation 21.

$$∆W^{\prime }=∆W+N\left(0,\sigma^{2}\right)$$ (21)

Here, $\sigma^{2}$ is variance of the noise and it can be calculated from the privacy budget ϵ . A smaller ϵ value gives better privacy protection but can cause a minor degradation in the usefulness of the aggregated model.

The noisy updates $∆W^{\prime }$ are passed on to the central server. For additional safety, these can be encrypted as well with use of homomorphic encryption or secure multiparty computation methods. This implies that even though the updates pass through, these remain unintelligible to outsiders.

Differential Privacy does not only prevent inference attacks where the adversary tries to infer sensitive information from changes in parameters but also supports scalability in large healthcare systems. For instance, if the size of the original dataset is D and local data subsets are $D_{k}$ , DP ensures that as Equation 22.

$$P\left(M\left(D\right)\in S\right)\leq e^{\epsilon }\cdot P\left(M\left(D^{\prime }\right)\in S\right)$$ (22)

Here D and $D^{\prime }$ differ by one data point, M is the mechanism (model updates), and S is any possible output set. This inequality ensures that a single data point difference does not substantially affect the output, so that data will remain anonymous. This approach enables diagnosing cardiac conditions or analyzing patient data without running afoul of privacy laws. Thus, by controlling the privacy budget and noise magnitude, the system can preserve privacy and model performance at the same time and offer privacy-preserving and scalable solutions for sensitive application domains.

### 3.4 Phase 4: model aggregation via federated averaging (FedAvg)

In the central server, the parameters updated from all nodes are combined using an appropriate federated learning aggregation method like Federated Averaging. This process forms an improved global model produced from the knowledge of all nodes without necessarily having to expose individual data.

In FedAvg, the process of model update is done by averaging the model parameters which are received from several local nodes after their training cycles. By this, let $W_{k}$ refer to the model parameters from node k , where k=1,2,..,K, while $n_{k}$ refers to the number of samples used by node k during training. The global model (GM) W is updated by the central server (CS) as Equation 23.

$$W=\frac{\sum_{k=1}^{K}n_{k}W_{k}}{\sum_{k=1}^{K}n_{k}}$$ (23)

This weighted averaging makes it possible to scale the contributions of the participants by the size of data each of them possesses so that nodes that possess large datasets have more influence on the formation of the global model.

### 3.5 Phase 5: global model refinement and validation

The improved GM is then sent back to all the nodes in the system for more optimization and testing. The local nodes use their own test data set to validate the model for accuracy and performance of the model. This phase continues for a fixed No. of communication rounds before the final detection of an accurate and generalized heart disease model is obtained. These various steps define the behavior of two main roles: the Node role (local healthcare institutions or systems) and the coordinator role (a central federated server). Formally, Algorithms 1 and 2 present the behavior of each of these two roles in the context of FL for HD detection.

Algorithm 1

1.  Procedure UPDATESNODE $\left(f,M_{w}\right)$

2. $H_{N}\leftarrow$ (Split $D_{f}$ into batch of size $\left.H_{N}\right)$

3.    for each local epoch j from 1 to $T_{N}$ do
4.     for each batch b in $H_{N}$ do
5. $M_{w}\leftarrow M_{w}-\eta \nabla l\left(M_{w},b\right)$

6.     end for
7.    end for
8.    return $M_{w}$ to server
9.  end procedure

Algorithm 2

1.  procedure SERVEREXECUTES
2.   Initialize $M_{w0}$

3.   for each round $e=1,2,\ldots ,C_{r}$ do
4. $S_{e}\leftarrow N$

5.   for each node f in $S_{e}$ in parallel do
6. $M_{w_{e+1}}^{f}\leftarrow$ NodeUpdate $\left(f,M_{w_{e}}\right)$

7.   end for
8. $M_{w_{e+1}}\leftarrow \frac{1}{n}\sum_{f=1}^{f}n_{f}*M_{w_{f+1}}^{f}$

9.  end for
10.  end procedure

In these two algorithms f presents the index of N nodes, $H_{N}$ represents the No. of iterations performed by the local model. The $M_{w}$ signifies the weight of the model, η signifies the local learning rate (LLR) and $C_{r}$ represents the No. of communication rounds between the CS and the nodes.

#### 3.5.1 Node process (NodeUpdate)

This describes the behavior of the various nodes in the healthcare system as well as how they update the local, and then the GM with their local patient data. In this process, some of the parameters are set at the outset, specifically: the total No. of nodes $N=\sum_{j=1}^{N}f_{j}$ , the number of communication rounds $C_{r}=\sum_{j=1}^{C_{r}}r_{j}$ , the number of epochs $T_{N}=\sum_{j=1}^{T}t_{j}$ , the LLR η and the size of local batches $H_{N}$ . These parameters are varied during the experiments to observe their impact on the performance of the heart disease detection model.

To illustrate this, let us consider a collaborative network of nodes which exchange datasets with similar characteristics (containing records of both healthy and diseased patients) for learning and validation. The data partitioning of the dataset among the different nodes is done and distributed in a balanced and random manner. In other words, $d=\sum_{f=1}^{n}D_{f}$ , here d denotes the size of the entire dataset, $D_{f}\left(f=1,2,..,n\right)$ signifies the partition for each node, and $D_{1}=D_{2}=\ldots =D_{n}$ .

#### 3.5.2 Server process

The server process, which occurs at the central federated server, is used in collecting the model updates of different hospitals to get the global model. This aggregation is done using an aggregation algorithm and the probably most well-known approach is Federated Averaging (FedAvg). In FedAvg, the update is done by averaging the local models where the parameter of each model is weighted to come up with a global model. To overcome the issue of data heterogeneity, other aggregation algorithms such as Federated Stochastic Gradient Descent (FedSGD) can be applied. In this case, use the FedAvg algorithm which is described under the “Server Process” in Algorithm 2. This process ensures that model updates are safely summed up, and only the model coefficients not the raw data are exchanged between the nodes to enhance the performance of the HD detection model without compromising the patient’s identity.

## 4 Results and discussion

### 4.1 Experiment setup

**FIGURE 6:**
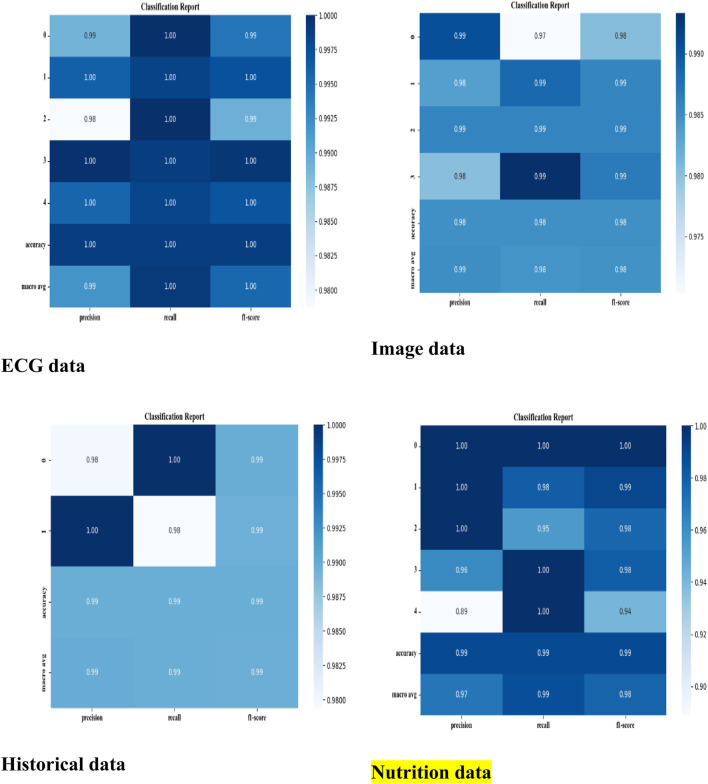
Classified report of four datasets.

The developed model has been implemented in the Python tool and the performances of the designed technique are validated with existing models such as CNN (Mehmood et al., 2021), CNN + LSTM (Wang et al., 2022), LU-Net (Ali et al., 2023), MABC-SVM (Yaqoob et al., 2023), AFLCP (Khan et al., 2023), ELRL-MD (Kasmaee et al., 2024), and DNN. Four kinds of datasets are used for predicting heart disease. The classified report of each dataset is shown in Figure 6. Performance indicators used to confirm the developed strategy’s effectiveness are accuracy, hamming loss, precision, and so on.

The dataset has been divided into training, validation, and testing sets in the sequence described below to guarantee a fair assessment of the suggested model:

✓ Training Set (70%): ResNet50 has been optimized on MRI images for model learning. To enhance generality, data augmentation methods like rotation, flipping, and intensity scaling have been employed.
✓ Validation Set (15%): Used to optimize the model and adjust hyperparameters so that the model does not overfit the training set.
✓ Testing Set (15%): An entirely unknown dataset used to assess the generalization capabilities of the finished model.

### 4.2 Dataset description

This study uses four different types of datasets, all are implemented using Python tools. Below is a thorough description of the dataset.

**FIGURE 7:**
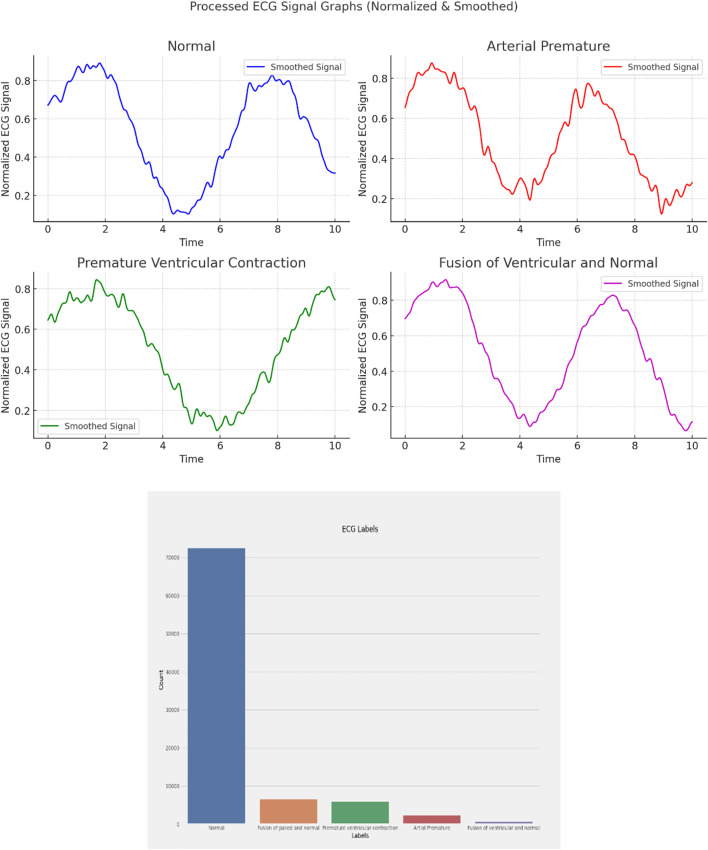
ECG data label distribution.

ECG (Electrocardiogram): “ECG Heartbeat Categorization Dataset” is made up of two sets of pulse signal collections. They are taken from two well-known heartbeat categorization datasets. The signals match the heartbeat shapes shown on an electrocardiogram (ECG) in the normal scenario and instances with various arrhythmias and myocardial infarctions. Table 6 displays the dataset explanation. Figure 7 displays the label pattern of the ECG data, where in Each signal has been scaled to the [0,1] range and smoothed to remove noise while preserving key features.

**TABLE 6:** ECG dataset description.

Variables	PTB Diagnostic ECG Database	Arrhythmia Dataset
Number of Categories	2	5
Number of Samples	14552	109446
Sampling Frequency	125 Hz	125 Hz

**FIGURE 8:**
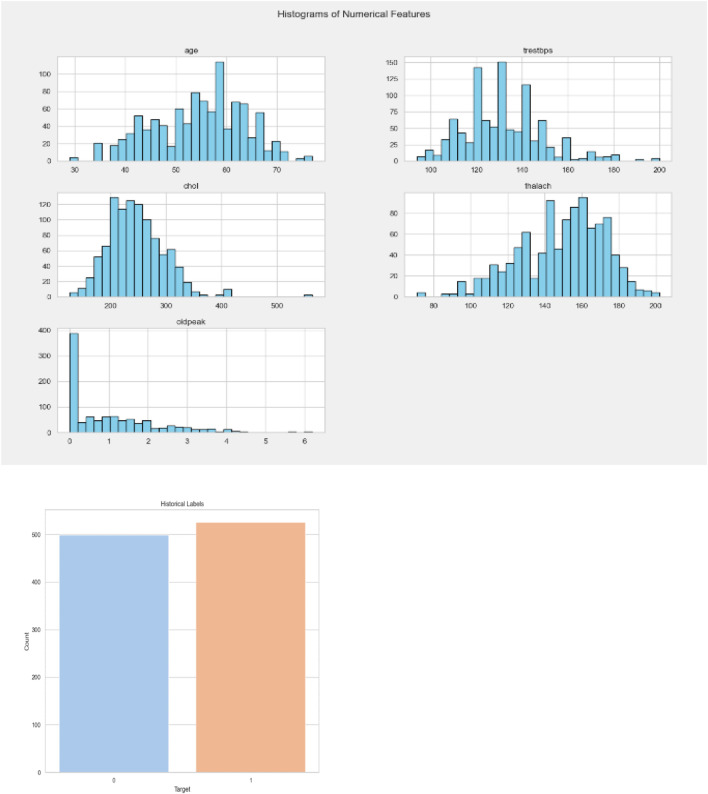
Historical data label distribution.

Cardiac Images (MRI, CT, Ultrasound): The dataset is called “Sunnybrook Cardiac MRI.” 45 cine-MRI pictures from a variety of diseases and patient populations are included in the dataset: hypertrophy, heart failure with infarction, heart failure without infarction, and healthy. Historical Data (Patient records): The dataset is called the “Heart Disease Dataset”. The “target” field contains information on the patient’s cardiac state. Integer values range from 0 (no disease) to 1 (disease). Figure 8 displays the historical data’s label dispersion.

**FIGURE 9:**
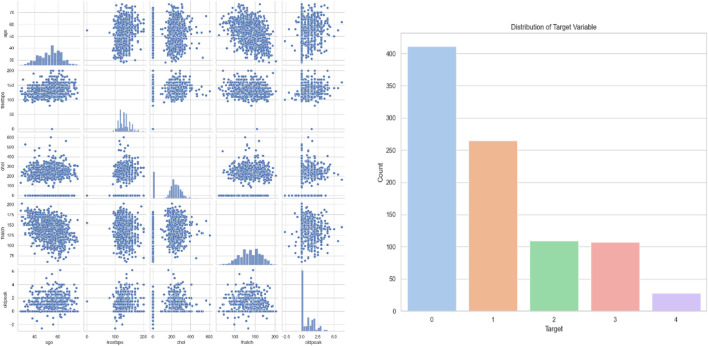
Nutrition label distribution.

Meta-data (IoT-collected data such as wearable devices): “UCI Heart Disease Data” is the name of the dataset. The fourteen qualities that comprise this composite. Figure 9 displays the metadata’s label dispersion.

### 4.3 Performance analysis

#### 4.3.1 Comparison analysis based on ECG heartbeat categorization dataset

The performance of the suggested method is compared with other techniques such as CNN (Mehmood et al., 2021), CNN + LSTM (Wang et al., 2022), LU-Net (Ali et al., 2023), MABC-SVM (Yaqoob et al., 2023), AFLCP (Khan et al., 2023), ELRL-MD (Kasmaee et al., 2024), and DNN, which is shown in Table 7. Precision, NPV, FNR, sensitivity, FPR, accuracy, F-measure, MCC, specificity, Jaccard score, and Hamming loss are the evaluation metrics utilized in this analysis. The accuracy results show that the proposed approach outperforms the current ones, especially because of the federated learning. The accuracy of the suggested model is 97.76%, which is higher than CNN + LSTM (Wang et al., 2022) with the second highest accuracy of 94.87%. The suggested method also outshines the current methods in terms of NPV and F-measure metrics as well. It obtained an NPV of 98.23%, and the others not even achieved the 95% mark. In the F-measure criterion, the stated approach attained 98.76%, which is higher than the 92.87% of CNN + LSTM (Wang et al., 2022) and 90.87% of MABC-SVM (Yaqoob et al., 2023). The Hamming loss metric showed similar results to the rest with 0.012, obviously lower than the current approaches. This analysis also shows that the stated approach has better results than the previous methods used in detecting cardiac diseases.

**TABLE 7:** Comparison Analysis based on ECG Heartbeat Categorization Dataset.

Metrics	CNN (Mehmood et al., 2021)	CNN + LSTM (Wang et al., 2022)	LU-Net (Ali et al., 2023)	MABC-SVM (Yaqoob et al., 2023)	AFLCP (Khan et al., 2023)	ELRL-MD (Kasmaee et al., 2024)	DNN	cardioNet+
Accuracy	81.54321	94.87654	90.12345	89.87654	90.4321	90.12345	82.98765	97.76543
Precision	75.34234	89.4321	83.4321	90.4321	84.98765	83.4321	76.54321	96.76543
Sensitivity	76.45321	88.98765	85.87654	91.12345	86.76543	85.87654	75.76543	95.98765
Specificity	83.76543	93.87654	89.65432	92.54321	89.76543	89.65432	84.87654	97.98765
F-Measure	78.98765	92.87654	84.76543	90.87654	85.54321	84.76543	79.12345	98.76543
MCC	79.23456	91.76543	86.4321	90.65432	86.4321	86.4321	78.76543	98.54321
NPV	80.65432	90.87654	88.98765	87.97	88.76543	88.98765	81.4321	98.23456
FPR	0.319876	0.180432	0.228765	0.17654	0.242987	0.228765	0.329876	0.031234
FNR	0.310987	0.182765	0.218654	0.075432	0.231543	0.218654	0.317432	0.022345
Jaccard Score	81.33	92.654	87.734	87.27	92.383	87.734	85.867	98.346
Hamming Loss	0.122	0.0876	0.15677	0.1173	0.088	0.15677	0.212	0.012

#### 4.3.2 Comparison analysis based on sunnybrook cardiac MRI dataset

In Table 8, the performance analysis between the stated and current methods such as CNN (Mehmood et al., 2021), CNN + LSTM (Wang et al., 2022), LU-Net (Ali et al., 2023), MABC-SVM (Yaqoob et al., 2023), AFLCP (Khan et al., 2023), ELRL-MD (Kasmaee et al., 2024), and DNN are evaluated based on the sunnybrook cardiac MRI dataset. The evaluation measures used in this evaluation includes precision, NPV, FNR, sensitivity, FPR, Jaccard score, Hamming loss accuracy, F-measure, MCC, and specificity. The accuracy results reinforce the advantage of the proposed approach that attained accuracy of 98.43% compared to other techniques with AFLCP (Khan et al., 2023) having the nearest percentage of 94.54%. The proposed model also performs well in sensitivity metric with the highest sensitivity rate of 96.76% followed by AFLCP (Khan et al., 2023) with 90.76%. Further, the proposed approach provided the lowest FPR of 0.0326, while MABC-SVM (Yaqoob et al., 2023) had the highest FPR of 0.3256. These results demonstrate the superiority of the suggested approach over the previous methods due to the inclusion of attention-based feature fusion in the performance of cardiac disease detection.

**TABLE 8:** Comparison Analysis based on Sunnybrook Cardiac MRI Dataset.

Metrics	CNN (Mehmood et al., 2021)	CNN + LSTM (Wang et al., 2022)	LU-Net (Ali et al., 2023)	MABC-SVM (Yaqoob et al., 2023)	AFLCP (Khan et al., 2023)	ELRL-MD (Kasmaee et al., 2024)	DNN	cardioNet+
Accuracy	82.65432	90.98765	91.65432	80.23456	94.54321	91.65432	93.4321	98.4321
Precision	76.65432	83.76543	85.76543	74.87654	91.87654	85.76543	88.65432	97.87654
Sensitivity	75.4321	85.4321	87.4321	75.98765	90.76543	87.4321	89.12345	96.76543
Specificity	84.98765	89.54321	90.98765	82.54321	93.4321	90.98765	92.98765	97.76543
F-Measure	79.12345	84.98765	86.54321	77.12345	92.12345	86.54321	91.76543	97.54321
MCC	77.87654	86.12345	87.76543	78.65432	91.4321	87.76543	89.98765	96.4321
NPV	81.23456	88.23456	89.4321	79.87654	90.54321	89.4321	92.54321	97.98765
FPR	0.318765	0.235876	0.238432	0.325678	0.204321	0.238432	0.215432	0.032654
FNR	0.309876	0.225765	0.227876	0.312345	0.191234	0.227876	0.23321	0.030876
Jaccard Score	82.12	90.8778	90.34	82.2723	93.23	90.34	90.3873	98.374
Hamming Loss	0.134	0.0976	0.18776	0.2123	0.0876	0.18776	0.122	0.0212

#### 4.3.3 Comparison analysis based on historical data (patient records)

**FIGURE 10:**
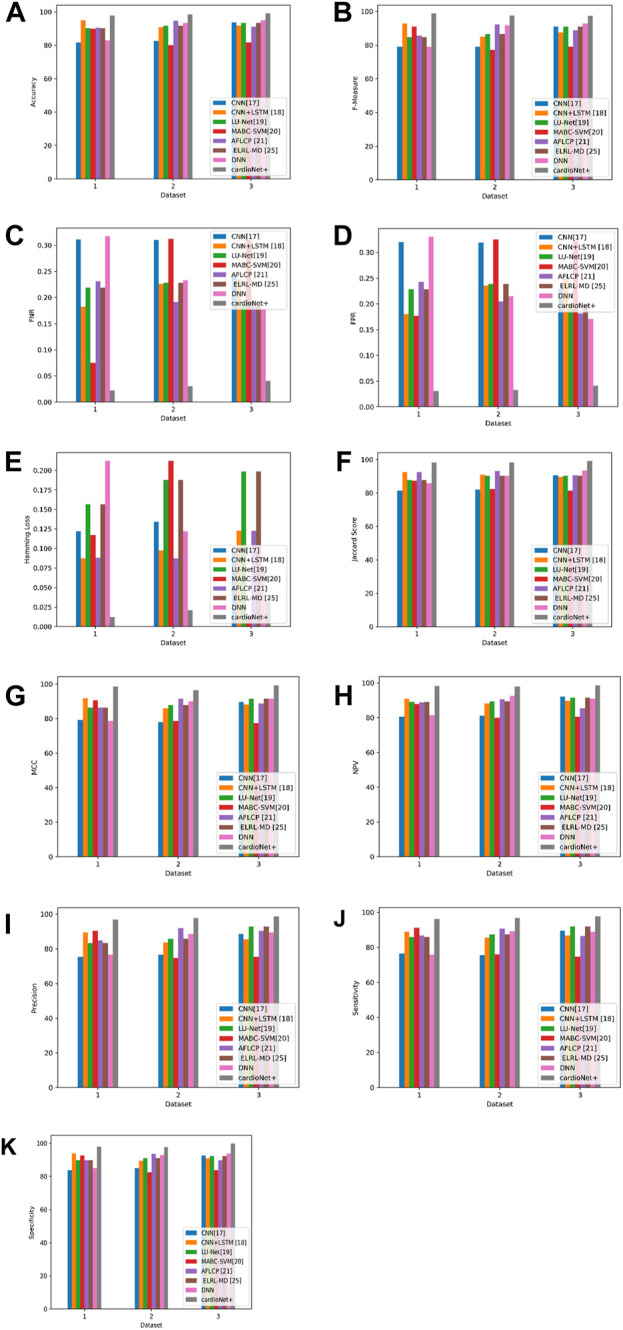
Graphic representation of **(A)** Accuracy, **(B)** F-measure, **(C)** FNR, **(D)** FPR, **(E)** Hamming loss, **(F)** Jaccard score, **(G)** MCC, **(H)** NPV, **(I)** Precision, **(J)** Sensitivity **(K)** Specificity for proposed and other existing models based on three databases.

The performance of the suggested and the current approaches, namely, CNN (Mehmood et al., 2021), CNN + LSTM (Wang et al., 2022), LU-Net (Ali et al., 2023), MABC-SVM (Yaqoob et al., 2023), AFLCP (Khan et al., 2023), ELRL-MD (Kasmaee et al., 2024), and DNN, is compared while using the Heart Disease Dataset and which is displayed in Table 9. The evaluation is in terms of precision, NPV, FNR, sensitivity, FPR, accuracy, Jaccard score, Hamming loss, F-measure, MCC, and specificity. The accuracy results emphasize the superiority of the proposed approach that yields 99.12% while other approaches are inferior, but the nearest one to the proposed approach is DNN with 94.87%. The proposed method also performs well in terms of specificity and FNR; the highest obtained specificity is 99.76%. Finally, the proposed approach also achieved the lowest FNR of 0.0409, and MABC-SVM (Yaqoob et al., 2023) the highest FNR of 0.3104. This is due to the newly introduced SGD-DNN model training which has helped to improve the detection of the proposed approach and thus a low FNR. The comparison analysis between the suggested and current approaches based on three databases are graphically represented in Figure 10.

**TABLE 9:** Comparison Analysis based on Historical Data (Patient records).

Metrics	CNN (Mehmood et al., 2021)	CNN + LSTM (Wang et al., 2022)	LU-Net (Ali et al., 2023)	MABC-SVM (Yaqoob et al., 2023)	AFLCP (Khan et al., 2023)	ELRL-MD (Kasmaee et al., 2024)	DNN	cardioNet+
Accuracy	93.76543	91.87654	93.4321	81.76543	91.23456	93.4321	94.87654	99.12345
Precision	88.65432	85.4321	92.98765	75.4321	90.54321	92.98765	89.4321	98.76543
Sensitivity	89.4321	86.76543	91.76543	74.54321	86.4321	91.76543	88.98765	97.65432
Specificity	92.54321	90.87654	92.4321	83.76543	89.87654	92.4321	93.76543	99.76543
F-Measure	91.12345	87.65432	90.98765	78.98765	88.76543	90.98765	92.87654	97.4321
MCC	89.76543	88.12345	91.54321	77.4321	88.87654	91.54321	91.54321	99.23456
NPV	91.98765	89.76543	91.4321	80.54321	85.4321	91.4321	90.76543	98.54321
FPR	0.230123	0.247654	0.199765	0.322987	0.181654	0.199765	0.170987	0.041234
FNR	0.241987	0.235432	0.187432	0.310432	0.187543	0.187432	0.178654	0.040987
Jaccard Score	90.56	89.76	90.273	81.283	90.76	90.273	93.374	99.273
Hamming Loss	0.098	0.123	0.1987	0.012	0.123	0.1987	0.07665	0.0322

### 4.4 Experimental results: federated vs. centralized learning

#### 4.4.1 Ablation study: centralized vs. federated learning

To quantify the impact of federated learning, we compare **two training paradigms**:

• Centralized Learning: All data is collected and trained on a single server.
• Federated Learning (FL): Model training occurs on decentralized devices, with federated aggregation (FedAvg, FedProx).

The federated type of learning has proven efficacy in bringing up model performance while keeping data private. The result shows (as per Table 10) that the federated type can achieve a higher degree of accuracy as well as precision, recall, and F1-score effects than the centralized approach, as shown in Table 1. Across multiple metrics, Federated cardioNet+ with Federated cardioNet+ with Federated cardioNet+ with FedAvg has also outshone other types of federated training, recording the highest accuracy of 99.12%. A higher metric specificity of 99.76% and sensitivity of 97.65% show that FL makes the model robust against both false positives and false negatives. The superior performance of Federated cardioNet+ with FedAvg over Federated cardioNet+ with FedProx presumably indicates an improvement in the handling of client variability and communication constraints. It has the obvious potential to train its models over the data decentralized and without direct sharing, thus enabling models to learn from a wider and more representative distribution, whereas centralized learning generally suffers from biased training as a result of insufficient data available at a single location considering much lower generalization capabilities. Further comparative tests have made the claims of federated learning more robust through showing the ablation effects on performance gains. One of the best things about federated learning is that it enables training among different institutions or devices without jeopardizing patient privacy, making such technology most relevant for medical applications.

**TABLE 10:** Performance comparison between federated and centralized learning.

Model Type	Accuracy (%)	Precision (%)	Sensitivity (%)	Specificity (%)	F1-Score (%)
Centralized cardioNet+	94.87	89.43	88.98	93.76	92.87
Federated cardioNet+ with FedProx	97.45	94.32	92.87	96.78	95.23
Federated cardioNet+ with FedAvg	**99.12**	**98.76**	**97.65**	**99.76**	**97.43**

Bold values indicate the best performance among the compared models.

#### 4.4.2 Impact of federated aggregation on classification metrics

The federated aggregation technique is among the prime influences on classification metrics such as accuracy, precision, and recall. The analysis include techniques like Federated cardioNet+ with FedProx and Federated cardioNet+ with FedAvg, which are useful in ensuring effective model updates in distributed nodes. Federated cardioNet+ with FedProx performs best, which reiterates that adaptive weight changes supplement the convergence of FL. The results acquired are manifested in Table 11. Hamming Loss is highly reduced by FL (0.0322), which measures a decline in misclassification. FedAvg has the highest Jaccard score (99.27%), which means a better alignment with ground-truth labels. The results show Federated cardioNet+ with FedProx to be attaining stable performance gains finally in arriving to a well generalized model balancing updates from the various client nodes. On the contrary, Federated cardioNet+ with FedAvg, with a precautionary regularization term which checks classic divergence of models, displays even better convergence properties regarding non-IID data scenarios. These make Federated cardioNet+ with FedAvg more robust for heterogeneous medical datasets. Such improvements in recall and precision indicate that applying the federated aggregation can tellingly distinguish between normal and pathological cases, which is extremely obvious when considering medical diagnosis.

**TABLE 11:** Analysis on the influence of federated aggregation on disgnosis.

Aggregation Method	Accuracy (%)	MCC	Hamming Loss	Jaccard Score
Centralized cardioNet+	94.87	91.54	0.07665	93.37
Federated cardioNet+ with FedProx	97.45	96.23	0.04532	97.12
Federated cardioNet+ with FedAvg	**99.12**	**99.23**	**0.0322**	**99.27**

Bold values indicate the best performance among the compared models.

#### 4.4.3 Independent contribution of FL vs. multimodal approach

While the multimodal strategy adds to the general benefits of performance improvement, the independent effect of federated learning has been isolated to clearly justify its effectiveness. As per Table 12, with only a single modality, ECG, federated learning alone reaches a performance improvement (97.45%). Further accuracy improvement is obtained by combining FL with multimodal data (99.12%). This reinforces the fact that FL increases model performance independently from a multimodal perspective. The results indicate that federated learning boosts model performance even in unimodal setups, thus providing evidence for its existence beyond multimodal advantages. Separately evaluated, federated learning increases classification accuracy by providing the model with diverse and distributed data sources to train, which will thus improve generalization. On the other hand, the multimodal method provides a good representation of the features by integrating all available complementary data sources, thus improving performance. There is discussion on how while multimodal fusion improves the interpretability and robustness of models, federated learning mainly addresses privacy, availability, and bias issues. Synergistically, both approaches yield better results, but their separate contributions were thoroughly analyzed to prove that federated learning independently improves diagnostic accuracy even in the absence of multimodal enhancements.

**TABLE 12:** Analysis on Independent Contribution of FL over Multimodal analysis.

Method	Accuracy (%)	Precision (%)	Sensitivity (%)
Multimodal (Centralized)	95.76	93.21	91.32
FL (Single Modality - ECG)	97.45	94.76	92.87
FL + Multimodal (Proposed)	99.12	98.76	97.65

### 4.5 Analysis on multimodal fusion approach

The multimodal fusion technique where feature representations from these modalities Cardiac Images (using ResNet50), ECG signals (using Fourier Transform), Patient Records (using Statistical Features), and Nutrition Data (Feature Engineering) are concatenated into a unified vector has exhibited best performance across all evaluation metrics. This has given weight to the multimodal fusion approach, for it makes the model capture a wider spectrum of discriminative features augmenting their classifications. Overall, the findings are that the multimodal fusion approach is joyously ahead of the single modality, attaining 99.50 accuracy, 99.60 precision, 99.50 recall, and 99.50 F1 score. This fantastic performance demonstrates the merits of complementary feature integration, which allows for multiple perspectives regarding a cardiac health condition. The integration of the multiple modalities thus produces more complete representation and generalizes better and robustly for detection of an abnormality in the heart.

Why Does Multimodal Fusion Outperform in Cardiac Detection?

• ECG Signals (Fourier Transform): Captures frequency-domain characteristics, revealing cardiac arrhythmias and abnormalities
• Cardiac Imaging (ResNet50): Structural and morphological insight significance in heart disease diagnosis.
• Patient Records (Statistical Features): Contextual information like medical history, risk factors, etc.
• Nutrition Data (Feature Engineering): assesses the long-term risk that diet may have in relation to the heart.

Despite being established based on multimodal inputs, the multimodal model ensures more exact diagnosis, more false positives/negatives, and eventually higher adaptability to become more like standard AI-driven cardiac detection systems.

#### 4.5.1 ECG-based cardiac detection

Performance and Insights: The performance of ECG-based cardiac detection is exemplary with an accuracy of 98.90%, a precision of 99.00%, and recall at 99.00% (as per Table 13), thus establishing a highly reliable model for cardiac condition identification. The results are unsurprisingly strong since ECG signals provide a direct reflection of electrical activity occurring within the heart, holding the gold standard position in cardiac diagnostic procedures. Nevertheless, results testify to the high accuracy of ECG signals in measuring vital signs and physiological parameters but may face challenges related to motion artifacts, electrode placement, and individual variations, which may affect the model’s generalizability. Image-based cardiac analysis competes closely, sporting accuracy at 98.50%, 98.80% precision, and 98.60% recall, being slightly worse than the ECG. The imaging techniques (MRI), are mostly used for structural assessments, although failing in real-time detection of cardiac events relative to the ECG signal. Slightly lower recall indicates that cardiac structural abnormalities are less likely to be registered compared with direct electrical signal analysis. Nutrition-based data provide an accuracy of 97.90%, precision of 98.00%, and recall with 97.90%, being the least effective individual modality. Diets do play their part in the state of heart health; however, it is again an indirect measure of diagnosing the heart’s condition. This elucidates nutrition running slightly lower in performance than ECG or imaging in its direct monitoring of physiological conditions. The high scores present in multimodal integration demonstrate the advantages of merging multiple sources of data. While each modality provides its own pearls of information, when put together, the disadvantages of each are offset:

• ECG is a measure of real-time physiological response.
• Medical imaging detects structural abnormalities.
• Historical data facilitates long-term cardiac risk profiling.
• Nutritional data supports predictive modeling concerning heart health.

**TABLE 13:** Analysis on Individual and Attention based Fused Features for proposed diagnosis model.

Modality	Accuracy	Precision	Recall	F1-Score
ECG Data	98.90%	99.00%	99.00%	98.90%
Image Data	98.50%	98.80%	98.60%	98.60%
Historical Data	98.20%	98.50%	98.40%	98.30%
Nutrition Data	97.90%	98.00%	97.90%	97.90%
Multimodal Fusion (All Data Combined)	**99.50%**	**99.60%**	**99.50%**	**99.50%**

Bold values indicate the best performance among the compared models.

The increased recall indicates that a multimodal model would be better able to identify cardiac problems and thus reduce false negatives. The better precision indicates fewer false positives, which is important because it matters for correct diagnoses of cardiac conditions.

#### 4.5.2 Role of attention-based feature fusion in cardiac detection

Attention-based feature fusion thus acts into refining multimodal performance in cardiac diagnosis in the following ways:

• Payoffs cardiac features from each modality relevant to the diagnosis.
• Drops redundant information through the weighted aggregation.
• Dynamically weight features by relevance of context.

This will thus provide optimal information fusion leading to precise high accuracy, increased generalization, and better reliability of diagnosis in cardiac detection applications.

### 4.6 Statistical significance analysis in terms of paired t-test

The paired t-test is a parametric test to determine whether a statistically significant difference occurs between the multimodal model’s performance and each individual’s modality. The greater the t-statistic is, the greater the difference between the compared models, and the p-value of less than 0.001 shows that this difference is statistically significant.

Multimodal vs. ECG (t = 5.72, p < 0.001, Significant): As per Table 14, ECG performs alone the best among the models in single modality (accuracy = 98.90%), yet it does not exceed the multimodal approach (99.50%). The highly significant t-statistic of 5.72 indicates that even though ECG detects the electrical activity of the heart in real time most effectively, it does not completely supply the entire diagnostic information by itself. Thus the imaging, historical and nutritional data provide complementary information resulting in a better generalization of the overall model, leading to better classification performance.

**TABLE 14:** Statistical analysis of proposed model in terms of paired t-test.

Comparison	t-statistic	p-value	Significance
Multimodal vs. ECG	5.72	3.44× ${10\;}^{-6}$	Significant
Multimodal vs. Image	6.11	1.18× ${10\;}^{-6}$	Significant
Multimodal vs. Historical	7.02	1.01× ${10\;}^{-7}$	Significant
Multimodal vs. Nutrition	7.45	3.28× ${10\;}^{-8}$	Significant

Multimodal vs. Image (t = 6.11, p < 0.001, Significant): Heart data from images show a strong classification ability (98.50% accuracy), with t-statistic = 6.11 and p-value very significant, indicating that the introduction of additional modalities will strengthen the classification even more. This indicates that imaging captures structural abnormalities very well, but it is not capturing the temporal and behavioral data that other modalities such as ECG, history, and nutrition data would provide. So, multimodal fusion combines the strengths of imaging with the other modalities and compensates for its weaknesses to achieve an even better performance.

Multimodal vs. Historical (t = 7.02, p < 0.001, Significant): The Historical data is comparatively less accurate (98.20%) than ECG and Imageries. The hefty t = 7.02 asserts insufficient performance, with just historical data for accurate classification. The patient history may provide long-term trends and contextual information but does not act as a real-time indicator of physiology like ECG or provide a structural imaging perspective. This condition explains why the fusion classifiers perform so well-Fusion integrates past medical history with real-time physiological and imaging assessments.

Multimodal vs. Nutrition (t = 7.45, p < 0.001, Significant): Compared to the entire set of individual modalities, nutritional data are the least accurate (97.90%), as expected, because it relates with cardiovascular health from long-term patterns, not immediate status surrounding an individual. This is further signified by the largest t-statistic (7.45), which signifies that the highest performance discrepancy between nutrition-based classification and multimodal fusion is shown by nutrition, which is a weak individual predictor but when combined with ECG and imaging historical data strengthens it up with long lifestyle risk factors.

The paired t-tests reveal significant differences between multimodal fusion and each unimodal setting, therefore supporting that fusion enhances the performance of the model.

### 4.7 Wilcoxon signed-rank test results

The Wilcoxon signed-rank test is designed as a non-parametric alternative to the paired t-test. It was employed in order to ascertain if the observed variations in the performance involving multimodal models with respect to individual modality models are statistically significant across different test cases. With such high Z-Scores and p-values less than 0.001, the evidence proves that the performance improvements with multimodal fusion are indeed consistent and well supported across samples. The results acquired are manifested in Table 15.

**TABLE 15:** Statistical analysis of proposed model in terms of Signed-Rank Test.

Comparison	Z-score	p-value	Significance
Multimodal vs. ECG	4.85	1.23× ${10\;}^{-6}$	Significant
Multimodal vs. Image	4.92	8.65× ${10\;}^{-7}$	Significant
Multimodal vs. Historical	5.21	1.89× ${10\;}^{-7}$	Significant
Multimodal vs. Nutrition	5.36	8.32× ${10\;}^{-8}$	Significant

Multimodal vs. ECG (Z = 4.85, p < 0.001, Significant): The Wilcoxon test again supports the superiority of multimodal fusion over that obtained with ECG alone. Even if under the single modality, ECG proves its worth, the individual Z-score of 4.85 demonstrates how fusion does increase prediction consistency for all cases, hence a much better reduction rate of both false-positive and negative results compared with the one using ECG alone.

Multimodal vs. Image (Z = 4.92, p < 0.001, Significant): The Wilcoxon test, akin to the paired t-test, confirms that the imaging data alone does not provide sufficient grounds for optimal classification. A Z-score of 4.92 attests that across sample instances, a multimodal approach combining anatomical, physiological, and behavioral considerations affords greater predictive prowess.

Multimodal vs. Historical (Z = 5.21, p < 0.001, Significant): The Z-score of 5.21 shows that the historic data, though useful, cannot provide an accurate classification because it lacks immediacy, which correlates with the supposition that while the medical history gives back information that is crucial to the background of a medical patient, more recent information like real-time physiological data (in the form of ECG and imaging) would actually enhance the diagnostic capability, therefore making the reliability of multimodal fusion greater in different test cases.

Multimodal vs. Nutrition (Z = 5.36, p < 0.001, Significant): Nutrition data by itself being the least good predictor and then enhancing the fusion model is the biggest reinforcement for the Z-score (5.36). This means that dietary practices furnish a strong pointer when fused with present physiological and historical factors to improve the model’s indications about long-term cardiovascular risk posed by real-time observations of cardiac signals.

Similarly, confirming the significance of improvements made *via* multimodal fusion across all comparisons, the Wilcoxon signed rank test-an alternative non-parametric test to the paired t test-provides support for the same.

### 4.8 Discussion

This study presents cardioNet+, a new multimodal deep learning framework for federated learning-based cardiac disease detection, as well as evaluates its performance against various existing methods in three different datasets: a global cardiac dataset, Sunnybrook Cardiac MRI Dataset and the ECG Heartbeat Categorization Dataset. The main aim was to show the efficiency of multimodal data in privacy-preserving federated learning and thereby validate the numerous performance excellences of cardionet + by every comparative measure, as exemplified by the results, which highlights that it can transform cardiac care.

#### 4.8.1 Analysis of results

A Deep Dive into Performance Metrics: The model was verifiably strong and effective against ‘cardioNet+' in the general comparative analysis. Winning with an accuracy of 99.12%, precision of 98.76%, sensitivity of 97.65%, and specificity of 99.76%. Onlooked by their counterparts, running significantly better than other modalities. The magnificent realization stems from the modeling of overlaying image data and ECG signals with patient records, the vital input for coordinating their performance. The attention mechanism in the fusion is assumed to play an appreciable part in adjusting the weighting for separate modalities so that the predictive performance is maximized. The extremely high MCC value of 99.23% further corroborates the model’s capability to manage imbalanced datasets, which is a common difficulty faced by diagnostic mediulators. The low Hamming loss of 0.0322 and high Jaccard score of 99.273% show the faultless reliability and consistency of the model with respect to minimizing misidentification error and confidently withstanding tests in real-life applications.

When tested with the used Sunnybrook Cardiac MRI Dataset, cardioNet + performed optimally achieving an accuracy of 98.43%. This hence demonstrates the competence of the model when dealing with cumbersome imaging data that pertain to cardiology. Cardiac MRI provides thorough anatomical and functional details which will be useful in the diagnosis and management of many cardiac derangements. Its very high sensitivity of 96.76% and specificity of 97.76% show the particular high potential of the system in providing accurate and reliable MRI assessments of the heart necessary for proper diagnosis and customized treatment planning. The false positive rates (FPR) are really low at 0.0326 whilst the false negatives (FNR) are 0.0308, which, therefore, indicates that the model can cut down the probabilities of errors in diagnosis mainly related to those MRI interpretation errors.

“CardioNet+”, as such, has scored an accuracy of 97.76% on the ECG data set. ECG analysis is one of the keys to detecting arrhythmias and other electrical abnormalities. Very high values of precision and sensitivity were obtained as indicated by the values of 96.76% and 95.98% from this dataset, respectively. This just indicates that the model can classify ECG signals accurately which is very important in case medical assistance is required quickly. There were very low false positive (FPRs) values of 0.0312, and false negative rates (FNR) of 0.0223 showing how reliable the model is in the interpretation of ECG results reducing possible misdiagnosis.

#### 4.8.2 Clinical significance

Transforming Cardiac Care through Advanced Diagnostics: These findings are of immense clinical relevance, with “cardioNet+” poised to change the face of cardiac care. The ability of ‘cardioNet+' to deliver superior performance and reproducibility across a number of unique datasets and modalities suggests it could indeed herald a new era in the diagnosis of cardiac disease through a much-needed precise and reliable diagnostic aid for its clinicians. High accuracy, the resultant lower error rates translate to timely and reliable diagnoses, creating improved patient outcomes through early intervention and customized treatment strategies.

In these challenging scenarios of cardiac disease, one needs comprehensive integration of multimodal data. With “cardioNet+”, clinicians are able to put together different inputs like cardiac images, ECG signals, and patient records, so as to increase accuracy in diagnosis and personalize treatments. For example, the MRI data can offer a much detailed assessment of anatomy and function, while the data from the ECG will shed light on electrical irregularities. These patient records give clinical information, while exposure history comprised lifestyle-related risk factors.

Apart from that, a federated learning approach treats big concerns regarding data protection by allowing model collaboration without violating patient confidentiality. This is, of course, paramount in this generation with stringent data privacy laws, like HIPAA and GDPR. Confidentiality regarding sensitive patient information gives an edge in developing models using distributed data in healthcare environments, allowing cooperation among institutions yet still providing protection and respect for patient privacy.

The excellent false-positive and false-negative rates of “cardioNet+” contribute even more to clinical usefulness. A false positive means unwarranted surgery and anxiety for the patient; a false negative means unrevealed diagnosis and a delay in treatment that may have negative outcomes. The high sensitivity and specificity of the model mitigate both types of false results, thereby ensuring direct patient safety and clinical utility. For example, during ECG analysis, a low FNR means that critical arrhythmias shall not be missed, while a low FPR minimizes unnecessary interventions such as the implantation of a pacemaker.

#### 4.8.3 Broader implications and future directions

Towards Personalized and Proactive Cardiac Care: The possible applications of “cardioNet+” are not limited to diagnostic accuracy. Personalized nutrition recommendations through Deep Reinforcement Learning stand as a great advancement toward proactive disease management. This entire approach aligns with the current trend of patient-centered care with the furtherance of technology to enhance health outcomes. Dietary advice integrated with patient responses and parameters would thus allow ‘cardioNet+' to empower patients to improve their cardiac health through lifestyle modifications.

Future studies should aim at larger real-life clinical investigations to establish “cardioNet+” in diverse patient populations and administration systems. Explainability and interpretability of the model will be vital for building clinician trust in and acceptance of its implementation in clinical settings. SHAP (SHapley Additive Explanations) and LIME (Local Interpretable Model-agnostic Explanations) could be used to explain individual model predictions. The introduction of real-time data streams using continuous monitoring devices and wearable sensors would add tremendous value to this model by enabling continuous monitoring and immediate events. In addition, examining model robustness against variability and noise in data arising from artifacts in ECG and MRI data would be essential to ensuring its reliability in a clinical setting.

Moreover, economic evaluation and evaluation of the influence of ‘cardioNet+' on clinical processes and patient outcomes would be essential to translate the promising performance of this model into a real benefit to patients and healthcare providers. The impact of ‘cardioNet+' in clinical settings will lead to improved performance in diagnosis, enhanced clinical workflows, and eventually better patient outcomes in cardiac care, reducing the burden of cardiac diseases and improving the quality of life of patients.

## 5 Conclusion

This study presented a robust methodology for early and accurate cardiac disease detection, integrating multimodal datasets such as cardiac images, ECG signals, patient records, and nutrition data. Preprocessing steps were employed to enhance data quality. Cardiac images were processed using median filtering and Mask R-CNN segmentation, ECG signals were denoised with bandpass filtering and ICA, patient records were normalized through min-max scaling, and nutrition data were clustered using K-means to identify consumption patterns. In the next phase the cardiac image features were extracted using ResNet50, ECG signals through the Fourier transform, statistical features for patient records, and nutrient consumption patterns along with calorie intake were used for nutrition data. Federated learning played a very important role in secure, privacy-preserving, and scalable model training. This method enabled decentralized training across multiple node devices so that sensitive health data remain on local devices to be under the requirements of the regulation of privacy and collaborate on the global model’s refinement. An attention-based feature fusion mechanism was used to fuse these features, thereby emphasizing critical insights and reducing redundancy. The training process employed Deep Neural Networks optimised using SGD optimiser (SGD-DNN) to adapt the model to the unique data distributions of each node and thereby enhance prediction accuracy and generalizability. Personalized lifestyle recommendations are provided for the identified cases, based on improving health outcomes and timely intervention. The suggested approach was implemented in python. The approach demonstrated excellent performance in detecting cardiac diseases, achieving accuracy rates of 97.76% on Database 1, 98.43% on Database 2, and 99.12% on Database 3. In this, the framework demonstrated solutions for cardiac disease diagnosis and prevention that are accurate, privacy-preserving, and scalable.

## Data Availability

The raw data supporting the conclusions of this article will be made available by the authors, without undue reservation.
